# MicroRNA Gets a Mighty Award

**DOI:** 10.1002/advs.202414625

**Published:** 2025-01-21

**Authors:** Yu Li, Sijie Chen, Hai Rao, Shengjin Cui, Guoan Chen

**Affiliations:** ^1^ Department of Human Cell Biology and Genetics Joint Laboratory of Guangdong‐Hong Kong Universities for Vascular Homeostasis and Diseases School of Medicine Southern University of Science and Technology Shenzhen Guangdong 518055 China; ^2^ Department of Biochemistry Key University Laboratory of Metabolism and Health of Guangdong School of Medicine Southern University of Science and Technology Shenzhen Guangdong 518055 China; ^3^ Clinical Laboratory The University of Hong Kong‐Shenzhen Hospital Shenzhen Guangdong 518053 China

**Keywords:** biomarker, cancer, miRNA, RNA biology, therapeutic approach

## Abstract

Recent advancements in microRNAs (miRNAs) research have revealed their key roles in both normal physiological processes and pathological conditions, leading to potential applications in diagnostics and therapeutics. However, the path forward is fraught with several scientific and technical challenges. This review article briefly explores the milestones of the discovery, biogenesis, functions, and application for clinical diagnostic and therapeutic strategies of miRNAs. The potential challenges and future directions are also discussed to fully harness their capabilities.

## Graphical abstract

Recent advancements in microRNA (miRNA) research highlight their crucial roles in physiology and disease, suggesting its potential application in diagnostics and therapeutics. This review focuses on miRNA discovery, biogenesis and action, roles in physiological and pathological processes, and clinical applications while addressing scientific challenges and exploring future directions for miRNA research and application.

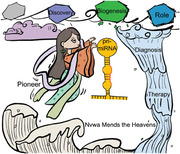

## 1 Introduction

MicroRNAs (miRNAs) are small, non‐coding RNA molecules that play a pivotal role in the regulation of gene expression at the post‐transcriptional level.^[^ ^1^ ^]^ Discovered in the early 1990s, miRNAs have since emerged as key players in a wide range of biological processes, including development, differentiation, and cellular response to environmental stimuli. These ≈22‐nucleotide‐long RNAs exert their effects primarily by binding to complementary sequences in the 3′ untranslated regions (3′ UTRs) of target messenger RNAs (mRNAs), leading to mRNA degradation or inhibition of translation.^[^ ^2^ ^]^ The intricate regulatory networks involving miRNAs have been implicated in various physiological and pathological conditions, such as cancer, cardiovascular diseases, and neurological disorders.^[^ ^3^ ^]^ Understanding the biogenesis, mechanism of action, and functional implications of miRNAs is essential for unraveling their contributions to gene regulation and their potential as therapeutic targets in disease management. This review aims to provide a comprehensive overview of the history of miRNA research over the years, miRNA biogenesis and action, and its broader roles across life science. We also discuss the implications of miRNA in diagnosis and therapeutics, as well as the challenges and future directions of miRNA research and application.

## 2 The Discovery of miRNAs

Recently, American scientists Victor Ambros and Gary Ruvkun were awarded the 2024 Nobel Prize in Physiology or Medicine for their discovery of miRNA.^[^ ^4^ ^]^ MiRNAs, a subset of non‐coding RNAs that typically contain ≈22 nucleotides, are endogenously produced short RNA molecules.^[^ ^5^ ^]^ Such non‐coding RNAs are believed to play a role in post‐transcriptional regulation by either facilitating the cleavage of targeted mRNAs or inhibiting their translation. In 1993, Victor Ambros discovered the first miRNA, lin‐4, in the *C. elegans*, and published his findings in the journal Cell.^[^ ^6^ ^]^ Intriguingly, around the same time, his colleague Gary Ruvkun uncovered the regulatory mechanism of lin‐14.^[^ ^7^ ^]^ Since the pioneering work of Ambros and Ruvkun, research on miRNA has been on the rise. In 2000, Gary Ruvkun discovered the second miRNA, named let‐7, in the *C.elegans*.^[^ ^2^ ^]^ Notably, let‐7 is highly conserved across animals, including in human cells. This groundbreaking discovery expanded the realm of miRNA research beyond nematodes to animals and ignited a surge of enthusiasm for studying miRNAs. In October 2001, three research groups led by Thomas Tuschl, David Bartel, and Victor Ambros, respectively, published articles in the same issue of the journal Science, naming this small RNA “microRNA”, abbreviated as miRNA.^[^ ^5^, ^8^ ^]^ Dicer, an enzyme that processes pre‐miRNA into mature miRNA, was reported by Mello et al. in 2001.^[^ ^9^ ^]^ In 2002, Croce et al. discovered the link between miRNA and disease.^[^ ^3b^ ^]^ Sam Griffiths‐Jones and colleagues established miRBase in 2002, with its first report published in 2004.^[^ ^10^ ^]^ MiRBase comprehensively collects and integrates miRNA sequences and standardizes their nomenclature and annotation, providing a centralized and reliable resource that has significantly enhanced the efficiency and quality of miRNA research. Drosha and Argonaute (AGO) were uncovered in 2003^[^ ^3c^ ^]^ and 2004.^[^ ^11^ ^]^ Next, major categories of conserved miRNA loci, functions, and their regulatory mechanisms have been discovered across different species, and attempts have been made to apply these findings in the diagnosis and treatment of human diseases.^[^ ^12^ ^]^ In 2008, Victor Ambros and Gary Ruvkun received the Albert Lasker Award for their discovery of miRNAs.^[^ ^13^ ^]^ Late, RNA‐binding proteins interacting with human pre‐miRNAs,^[^ ^14^ ^]^ and nuclear activating miRNAs (NamiRNAs) were uncovered in 2017.^[^ ^15^ ^]^ In 2018, Jee et al. reported that Ago2‐mediated miR‐486 biogenesis requires Dicer.^[^ ^16^ ^]^ In the same year, the CRISPR‐Cas9 platform is used in the miRNAs detection^[^ ^17^ ^]^ (**Figure** **1**A).

Figure 1Milestone discoveries in the history of miRNA research. A). The timeline of miRNA research from 1993 until 2018. It includes major events such as: **1993**: Discovery of the first miRNA (lin‐4) in C. elegans.^[^ ^6^, ^7^ ^]^ **2000**: Discovery of let‐7, the second miRNA, highly conserved across animals.^[^ ^2^ ^]^ **2001**: 1) miRNA officially named.^[^ ^5^, ^8^ ^]^ 2) Discovery of Dicer, an enzyme that processes pre‐miRNA into mature miRNA.^[^ ^9^ ^]^ **2002**: 1) Link between miRNA and disease is established.^[^ ^3b^ ^]^ 2) First report of miRBase, providing a comprehensive miRNA database for research.^[^ ^10^ ^]^ 3) miRNA base‐pairing with the 3′ UTR of mRNA.^[^ ^1a^ ^]^ **2003**: 1) Discovery of Drosha, an enzyme for the nuclear processing of primary miRNA (pri‐miRNA).^[^ ^3c^ ^]^ 2) The discovery of Exportin‐5 is specifically responsible for transporting pre‐miRNA from the nucleus to the cytoplasm.^[^ ^12a^ ^]^ 3) It was first reported that miRNA plays an important role in embryonic morphogenesis and differentiation.^[^ ^12e^ ^]^ **2004**: 1) Identification of Argonaute (AGO), a key component of the RNA‐induced silencing complex (RISC).^[^ ^11^ ^]^ 2) First report of miRNAs playing an important role in the regulation of metabolism in mammals.^[^ ^12b^ ^]^ **2005**: First oncogenic miRNAs are reported.^[^ ^12c,d^ ^]^ **2007**: 1) Drosha‐independent pathway of miRNA biogenesis‐ mirtron pathway.^[^ ^12f,h^ ^]^ 2) The first time that miRNA can be used as a medium of intercellular communication.^[^ ^12g^ ^]^ **2008**: 1) Drosha‐independent pathway of snoRNA/shRNA‐derived miRNAs.^[^ ^3^, ^26^ ^]^ 2) Victor Ambros, Gary Ruvkun, and David Baulcombe were awarded the Lasker Foundation for their discovery of miRNAs.^[^ ^13^ ^]^ 3) miRview mets‐the earliest miRNA diagnostic kit.^[^ ^12i^ ^]^ **2009**: The first miRNA inhibitor miravirsen entered human clinical trials.^[^ ^12j^ ^]^ **2010**: 1) Drosha‐independent pathway of tRNA‐derived miRNAs.^[^ ^28^ ^]^ 2) Ago2‐mediated cleavage of miRNA biogenesis that is independent of Dicer.^[^ ^29^ ^]^ **2013**: miRview mets received FDA approval. **2017**: 1) 180 RNA‐binding proteins specifically interact with human pre‐miRNAs.^[^ ^14^ ^]^ 2) miRNA promotes gene expression by acting on enhances as NamiRNA.^[^ ^15^ ^]^ **2018**: 1) Ago2‐mediated cleavage of miRNA biogenesis that also requires Dicer.^[^ ^16^ ^]^ 2) The first to use CRISPR‐Cas9 in miRNA detection.^[^ ^17^ ^]^ B). Recent advancements in miRNA research over the past five years. **2020**: DIANA‐miRGen v4 uniquely integrates cell‐specific miRNA promoters with experimentally derived TFBSs.^[^ ^18a^ ^]^ **2021**: 1) A molecular model explaining how polypeptides collaborate to accurately recognize and process pri‐miRNAs.^[^ ^23a^ ^]^ 2) A quantitative map of human primary microRNA processing sites.^[^ ^23b^ ^]^ **2022**: 1) The plasma miRNA profile at COVID‐19 and identify miRNAs as early prognostic biomarkers of severity and survival.^[^ ^21^ ^]^ 2) Reveal miRNAs encoded by herpesviruses selectively inhibit the miRNA processing of host cells.^[^ ^20^ ^]^ **2023**: 1) Cryo‐electron microscopy structure of hDICER bound to pre‐miRNA in a dicing state.^[^ ^19^ ^]^ 2) The developed Exo‐PROS biosensor achieves simultaneous detection of proteins and miRNAs in exosomes originating from tumors.^[^ ^22a^ ^]^ **2024**: 1) Victor Ambros and Gary Ruvkun were awarded the 2024 Nobel Prize in Physiology or Medicine for their discovery of miRNA.^[^ ^4^ ^]^ 2) A computational method based on multiple hypergraph contrastive learning (MHCLMDA) to predict miRNA–disease associations.^[^ ^3a^ ^]^ 3) The first comprehensive reference list of cancer‐related miRNA genes.^[^ ^23c^ ^]^ 4) A novel therapeutic approach merging miRNA regulation with PROTACs' protein degradation.^[^ ^18b^ ^]^ 5) Hypoxic glioma cells promote the M2 polarization of tumor‐associated macrophages by secreting exosomes rich in miR‐25‐3p.^[^ ^22b^ ^]^ C). Trends of global publications on the topic of miRNA. The data are obtained from the PubMed database using the search query “(miRNA) AND (microRNA)”.

**Figure:**
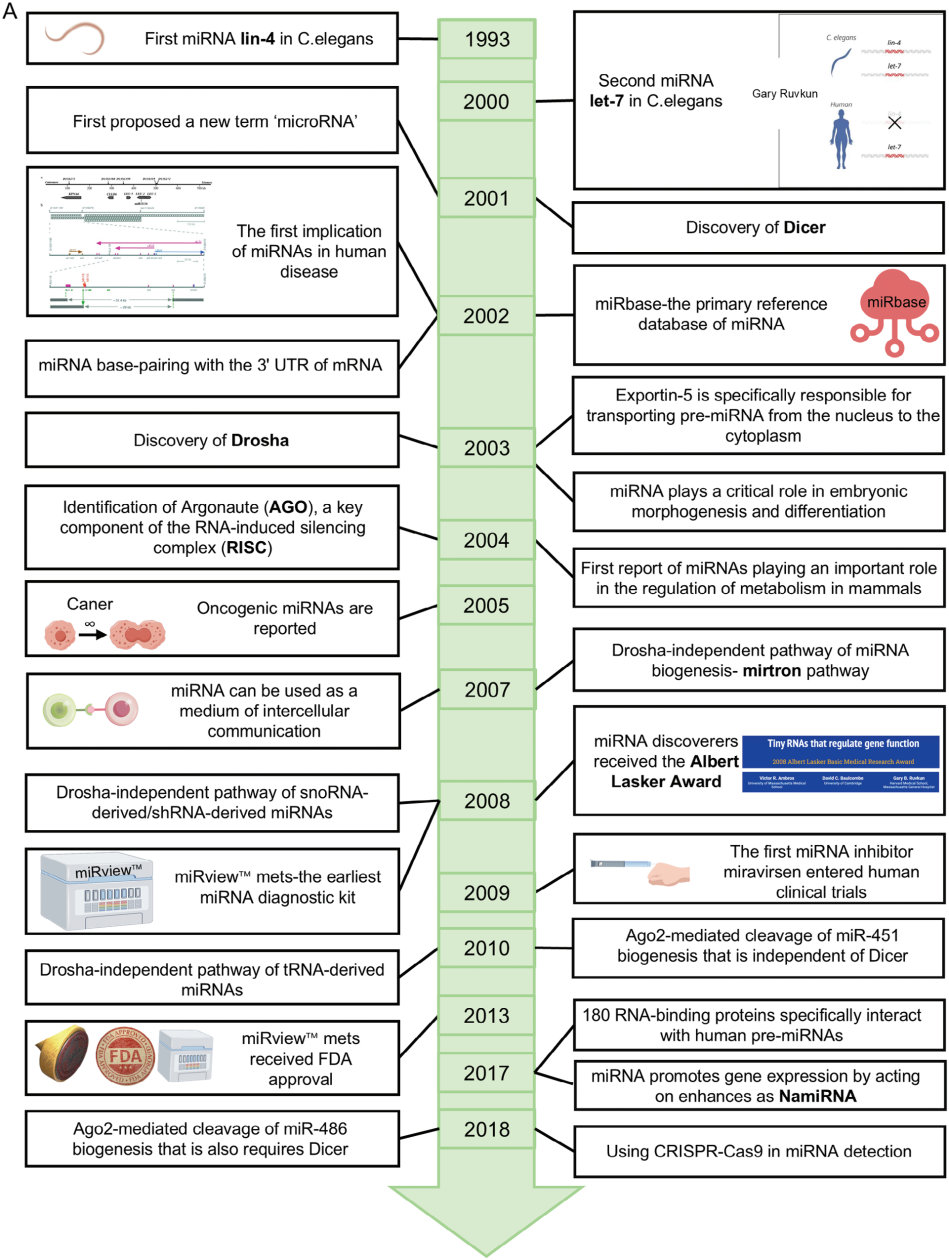

**Figure:**
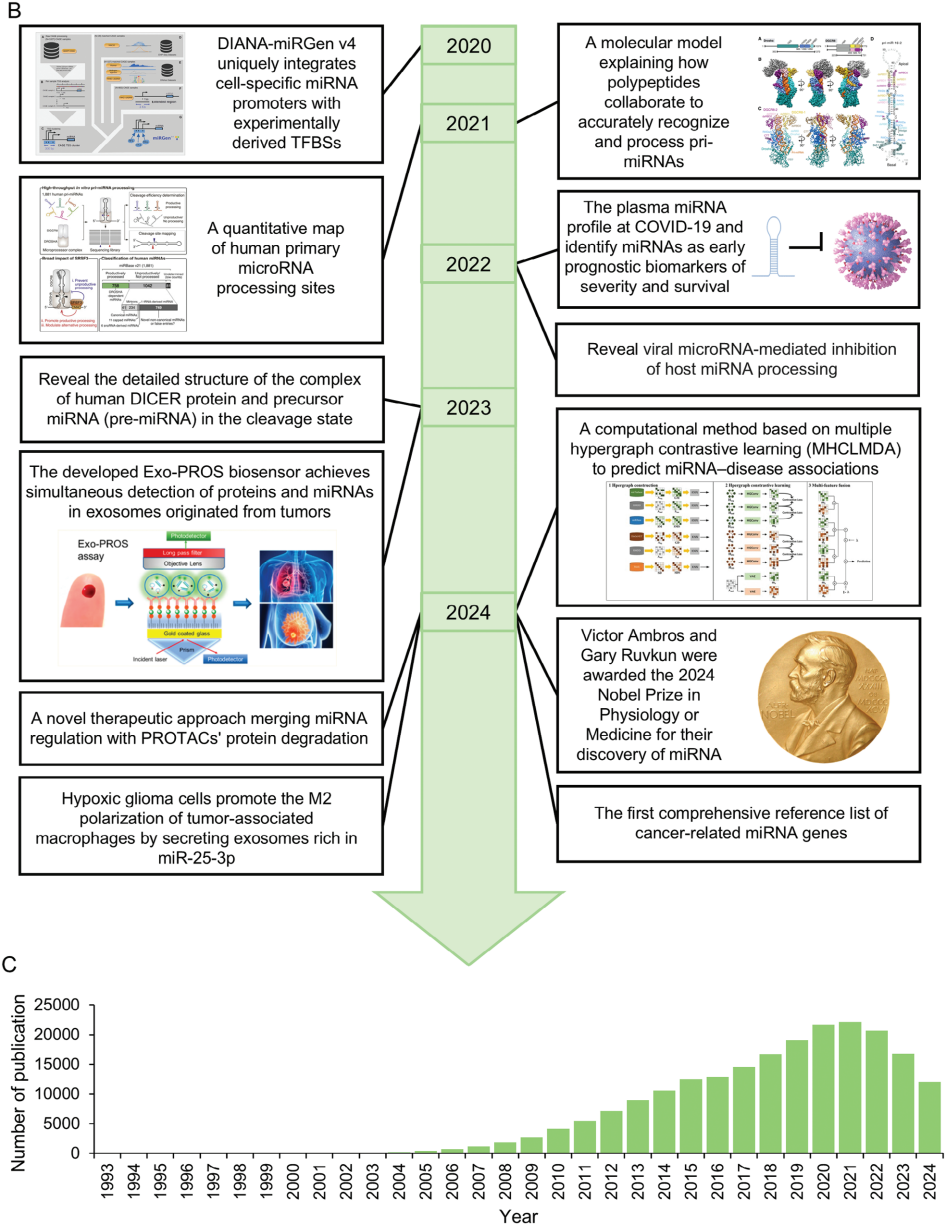

In recent years, there have been significant developments in miRNA research. Scientists have utilized bioinformatics methods to construct new regulatory networks for miRNAs and their associations with diseases.^[^ ^3^, ^15^, ^18^ ^]^ Advanced techniques such as cryo‐electron microscopy have allowed for the observation of the structures of critical enzymes involved in miRNA processing, such as Drosha and DICER.^[^ ^19^ ^]^ Interactions between viruses and miRNAs have been discovered.^[^ ^20^ ^]^ In addition, plasma miRNAs have been identified as potential biomarkers for COVID‐19,^[^ ^21^ ^]^ and exosome miRNAs are further explored and serve as cancer biomarkers.^[^ ^22^ ^]^ These studies highlight that, with ongoing technological advancements and the emergence of new techniques, miRNA research is likely to yield increasingly remarkable results^[^ ^23^ ^]^ (Figure 1B, C).

## 3 The Biogenesis and Action of miRNAs

**Figure 2:**
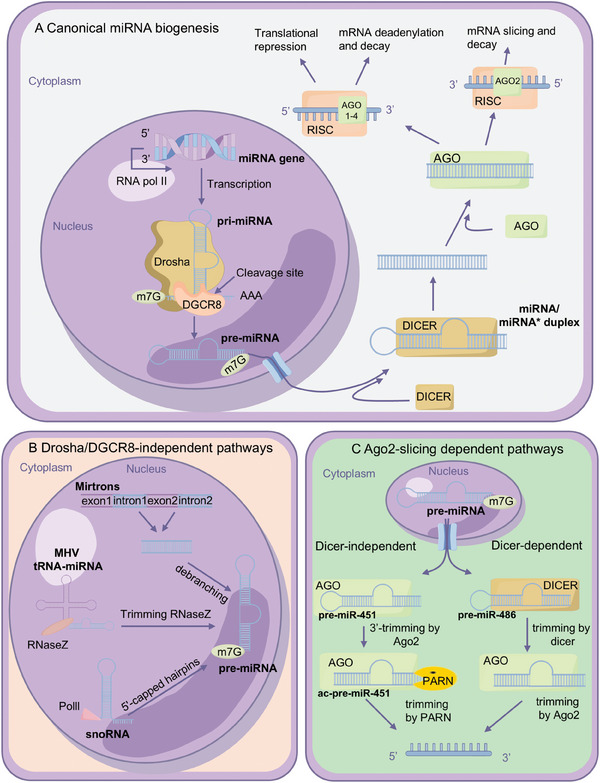
Canonical and major non‐canonical miRNA biogenesis pathways. A) MicroRNAs are produced from genes through a canonical pathway: they are transcribed into hairpin‐shaped primary miRNAs (pri‐miRNAs), cleaved by Drosha into precursor miRNAs (pre‐miRNAs), and exported to the cytoplasm by Exportin‐5. Dicer then processes them into duplex microRNA strands, one of which is incorporated into the RNA‐induced silencing complex (RISC) to interact with mRNA. B) Non‐canonical pathways (Drosha‐independent). (Top) Mirtron Pathway: Pre‐miRNAs are generated from spliced introns instead of being processed by Drosha. These intronic sequences fold into hairpin structures and are exported to the cytoplasm for Dicer processing. (Middle) tRNA‐Derived miRNAs: Transfer RNAs (tRNAs) can also be processed into small RNA molecules, bypassing Drosha in a similar fashion. (bottom) snoRNA‐Derived miRNAs: Small nucleolar RNAs (snoRNAs) can serve as precursors for miRNAs, bypassing Drosha cleavage but still requiring Dicer for maturation. C) Non‐canonical pathways (Ago2‐cleavage dependent). (Left) Pri‐miRNA‐451 is processed by Drosha into pre‐miRNA‐451, which is directly loaded onto Ago2. Ago2's slicer activity cleaves the 3′ arm, producing ac‐pre‐miRNA‐451, which then forms mature miR‐451. (Right) MiR‐486 has perfect base‐pairing in its pre‐miRNA stem and needs Drosha, Dicer, and Ago2 to cleave the passenger strand, forming the mature single‐stranded RISC. PARN: Poly(A)‐Specific Ribonuclease.

All microRNAs are generated from their respective genes via multi‐step processes. Initially, they are transcribed to form hairpin‐structured primary miRNAs (pri‐miRNAs) and then cleaved by the microprocessor complex with Drosha and Dgcr8 enzymes into precursor miRNAs (pre‐microRNAs).^[^ ^3c^ ^]^ After the export of pre‐miRNAs to the cytoplasm by Exportin‐5, Dicer subsequently processes the pre‐microRNAs, leading to the creation of duplex microRNA strands.^[^ ^9^, ^12^, ^24^ ^]^ One of these strands is then loaded into the RNA‐induced silencing complex (RISC, also named the functional Ago effector complex) where it interacts with mRNA^[^ ^11^, ^25^ ^]^ (**Figure** **2**A). In addition to this classical biogenesis pathway, there are several non‐canonical pathways, including Drosha‐independent (Figure 2B) and Ago2‐cleavage‐dependent (Figure 2C).

Drosha‐independent pathways of miRNA biogenesis are pre‐miRNA primarily produced by spliced introns (mirtron pathway),^[^ ^12f,h^ ^]^ snoRNAs,^[^ ^26^ ^]^ shRNA,^[^ ^27^ ^]^ or tRNAs.^[^ ^28^ ^]^ While the majority of miRNAs do not necessitate the catalytic activity of Ago2 for their biogenesis, there are two notable conserved exceptions. The most well‐known miRNA generated through Ago2‐mediated cleavage is miR‐451.^[^ ^29^ ^]^ Ago2 is the only member of the Argonaute family with nuclease activity. This miRNA plays a vital regulatory role in erythrocyte differentiation, and its biogenesis depends entirely on Ago2, rather than Dicer. Due to its short precursor length, miR‐451 is not suitable for Dicer recognition and is instead processed by Ago2.^[^ ^30^ ^]^ MiR‐486 exhibits perfect base‐pairing within its pre‐miRNA stem structure and necessitates the involvement of Drosha and Dicer enzymes for the generation of a miRNA duplex. Furthermore, the maturation into a single‐stranded RNA‐induced silencing complex (RISC) requires the cleavage of the passenger strand by Ago2.^[^ ^16^ ^]^

**Figure 3:**
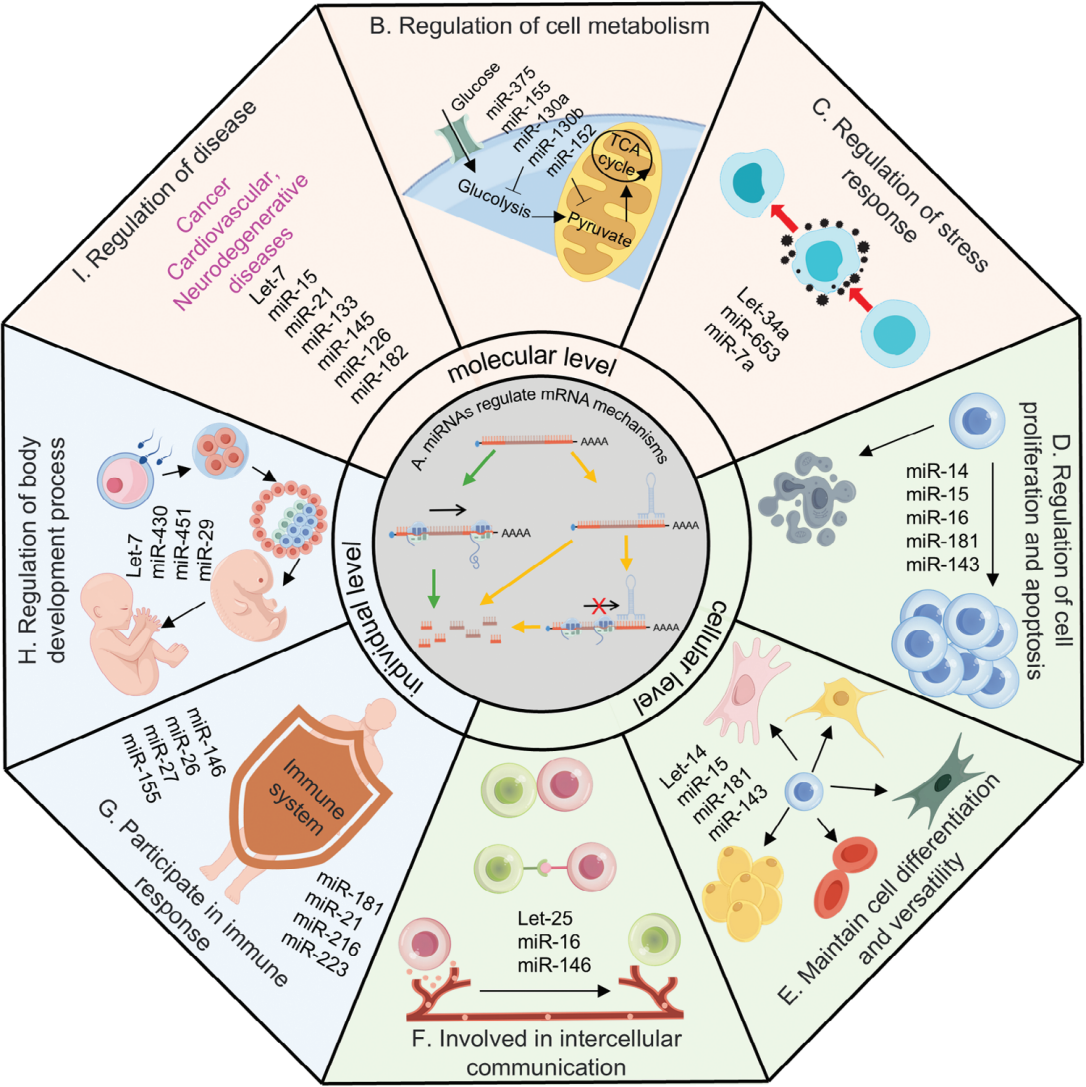
Regulation mechanism and function of miRNA. A) miRNAs regulate mRNA expression. (Left) The green arrow indicates that, in the absence of miRNA, mRNA can be efficiently translated into protein before undergoing degradation. (Right) The yellow arrow signifies that, in the presence of miRNA, the translation of mRNA is inhibited, or mRNA is degraded. B–I) miRNAs are involved in normal physiological processes and pathological conditions at different levels.

The action model of miRNA targeting depends on the base pairing between the seed region (nucleotides 2–7) of the miRNA and sites within the 3′ UTRs of mRNA.^[^ ^1^, ^31^ ^]^ Fully complementary binding occurs when the “seed” region of the miRNA binds to the target mRNA, resulting in the degradation of the mRNA by the slicing and decay process, which frequently happens in plants and lower eukaryotes. In contrast, imperfect base pairing leads to translational inhibition and deadenylation of mRNAs, which are the major model in animals^[^ ^1^, ^32^ ^]^ (Figures 2A and 3A). Additionally, miRNAs can act as “decoys” and interfere with the function of regulatory proteins by binding to mRNA without the need for seed region complementarity (Figure 2A).^[^ ^33^ ^]^ In recent studies, it has been demonstrated that miRNA binding sites also can be found in both 5′UTRs and coding sequences.^[^ ^32^ ^]^ Approximately 60% of protein‐coding mRNAs are regulated by miRNAs. A single miRNA may target hundreds of mRNAs, and each of those mRNAs may be targeted by several miRNAs in different regions. On the other hand, miRNA itself is modulated by a multitude of effectors when performing its basic functions, such as epigenetic mechanisms, cellular or environmental changes, miRNA editing, and circular RNA sponging.^[^ ^34^ ^]^

## 4 The Roles of miRNAs in Physiological and Pathological Processes

miRNAs are essential gene regulators that maintain cellular homeostasis and facilitate adaptation to environmental changes. Numerous studies emerge every year to elucidate the roles of miRNAs in both normal physiological processes and pathological conditions.^[^ ^35^ ^]^ The primary functions of miRNAs encompass the regulation of cellular metabolism, stress response, cell proliferation and apoptosis, cell differentiation and pluripotency, intercellular communication, immune responses, body developmental processes, and disease pathways, predominantly, through the negative regulation of gene expression^[^ ^36^ ^]^ (**Figure** **3**A–I).

### 4.1 Regulation of Cell Metabolism and Cell Stress Response

In 2004, Poy et al. reported that miRNA‐375 could regulate normal physiological metabolism via *Mtpn* (*Myotrophin*) to regulate insulin secretion^[^ ^12b^ ^]^ (Figure 3B). This not only was the first report on the function of miRNA in pancreatic endocrine cells but also provided the first clear experimental evidence that miRNA plays a significant role in the physiological processes of mammals in metabolic regulation. MiR155/miR‐143 cascade can upregulate *HK2* (*Hexokinase*) mRNA level through activation of STAT3 signaling pathway.^[^ ^37^ ^]^

miRNAs can not only regulate normal physiological and pathological metabolisms but also assist cells in resisting external stress. In 2010, Ito T et al. proposed the regulatory role of miR‐34a in oxidative stress‐induced endothelial cell senescence, suggesting that miRNAs have significant functions in the process of cells responding to oxidative stress^[^ ^38^ ^]^(Figure 3C). Nevertheless, the role of miRNAs in oxidative stress was gradually identified after a certain degree of development in the miRNA field. To this point, it has been gradually realized that miRNAs participate in regulating the responses of cells to external stimuli, such as oxidative stress. In‐depth analysis of the interaction between miRNAs and key signaling pathways, and revelation of the role of miRNAs in regulating responses to stress can provide a basis for identifying new therapeutic targets and developing targeted miRNA therapeutics. For instance, a recent study indicates that the miR‐653‐3p/IGF1 axis can serve as a novel therapeutic target for migraines.^[^ ^39^ ^]^ By inhibiting miR‐653‐3p, it can effectively alleviate NO₂‐induced migraines, providing a new idea for the future development of therapeutic approaches for neurological diseases induced by environmental pollutants. The research conducted by Yuan et al. shows that miR‐7a‐5p regulates the molecular mechanism of food intake in AgRP neurons,^[^ ^40^ ^]^ deepening the understanding of energy metabolism and appetite control and offering new insights for the treatment of related diseases.

### 4.2 Regulation of Cell Proliferation, Apoptosis, and Cell Differentiation

miRNA can also affect cell proliferation, apoptosis, and cell differentiation by regulating gene expression^[^ ^41^ ^]^ (Figure 3D, E). In 2003, a study by Xu et al. reported the Drosophila miR‐14 can act as a cell death suppressor to regulate cell apoptosis in multicellular organisms.^[^ ^42^ ^]^ While miR‐15 and miR‐16 can induce cell apoptosis by targeting the anti‐apoptotic gene BCL2 in human chronic lymphocytic leukemia,^[^ ^43^ ^]^ demonstrating that miRNA regulates cell apoptosis in human cells. MiRNA can also regulate cell differentiation. Chen et al. explored the role of miR‐181 in regulating the differentiation process of hematopoietic stem cells (HSCs).^[^ ^44^ ^]^ This article was the first to demonstrate that miRNA can regulate the lineage differentiation of cells in the mammalian hematopoietic system, enriching our knowledge of the mechanisms of cell differentiation and development, laying a foundation for further exploration of the role of miRNA in biological development and diseases, and providing a potential direction for the development of new therapeutic strategies. In the same year, Esau et al. revealed the regulatory role of miRNA‐143 in promoting adipocyte differentiation via target gene ERK5 (MAPK7, Mitogen‐activated protein kinase 7),^[^ ^45^ ^]^ further extending the role of miRNA in determining cell fate.

### 4.3 Involved in Intercellular Communication

MiRNA not only influences the cells themselves but also communicates with the surrounding cells and regulates them^[^ ^46^ ^]^ (Figure 3F). The earliest article demonstrating that exosome miRNAs can act as an intercellular communication was reported in 2007.^[^ ^12g^ ^]^ This article initially disclosed that exosomes contain functional mRNA and miRNA, including *let‐7, miR‐1, miR‐15, miR‐16, miR‐181*, and *miR‐375*, etc, and these molecules can be absorbed by other cells and translated or exert gene regulatory effects in recipient cells. In 2010, Kosaka, N et al. elucidated the secretion mechanism of miRNA, e.g. miR‐146a, in living cells,^[^ ^47^ ^]^ which shows how these miRNA molecules are transferred between cells via microvesicles. These results further confirm the role of miRNAs in cell communication, provide a basis for understanding the disease process, and help to develop new therapeutic strategies. Recent literature has reported that hypoxic glioma cells secrete exosomes enriched with miR‐25‐3p, promoting the M2 polarization of tumor‐associated macrophages.^[^ ^22b^ ^]^ This mechanism unveils the intricate interactions between tumor cells and immune cells, offering a novel potential target and thinking for the treatment of glioma. In conclusion, the regulation of miRNAs on cells themselves or on the surrounding cells is one of the hotspots in current life science research. The latest studies constantly deepen our understanding and reveal its significant role in both healthy and diseased conditions.

### 4.4 Participate in Immune Response

The role of miRNAs in regulating gene expression has been extensively investigated, however, research on the specific functions and mechanisms of miRNAs in the immune system has been relatively late.^[^ ^48^ ^]^ The earliest study that associated miRNA with the body's immune system was reported by Taganov et al. in 2006, which mainly uncovered the high expression of miR‐146 in macrophages and other immune cells, and its specific role in immune reactions.^[^ ^49^ ^]^ This study provided a solid theoretical foundation for subsequent research on the role of miRNAs in the immune system and promoted the development of the interdisciplinary field of miRNAs and immunology. Since the pioneering work of Taganov et al., the research on the role of miRNAs in the immune system has rapidly advanced in aspects of adaptive immunity, immune cell differentiation, regulation of inflammatory responses, and autoimmune diseases (Figure 3G). Recently, research conducted by Sarah Ressel et al. systematically analyzed the RNA‐RNA interactions between RSV and the host miR‐26 and miR‐27, uncovering how the virus exploits the miRNA network to regulate the host cell cycle and antiviral immune responses.^[^ ^50^ ^]^ This not only deepened our comprehension of the RSV infection mechanism but also provided a theoretical foundation and potential application directions for developing novel anti‐RSV therapeutic strategies. Particularly, the identification of miR‐26 and miR‐27 as key regulatory factors has paved a new way for virus infection intervention through the miRNA regulatory network.

### 4.5 Regulation of Body Development Process

MiRNAs can possess the property of regulating gene expression with spatiotemporal specificity, ensuring the timely expression of specific genes in different stages of embryonic development and different cell types, and guaranteeing cells following specific fates at specific times^[^ ^51^ ^]^ (Figure 3H). The earliest literature reporting the regulation of embryonic development by Dicer was published in 2003.^[^ ^12e^ ^]^ This pioneering study reported the central regulatory role of Dicer in embryonic development, laying the foundation for subsequent research on miRNAs in developmental biology. In 2005, the research by Giraldez et al.^[^ ^52^ ^]^ reported that miR‐430 regulates vertebrate organogenesis by rapidly degrading maternal mRNA, informing us that miRNAs play significant roles not only in the early stage of embryogenesis but also during the organ development phase.

### 4.6 Roles in Diseases

The roles of miRNAs in multiple diseases are extensively reported. By regulating gene expression, miRNA influences the pathological mechanisms of diseases including cancer, cardiovascular diseases, neurodegenerative diseases, leukemia, metabolic disorders, etc.^[^ ^53^ ^]^ (Figure 3I). One of the classic literature on which the role of miRNA in diseases was first reported by Calin et al. in 2002.^[^ ^3b^ ^]^ This study revealed that the deletion or down‐regulation of the miR‐15 and miR‐16 was closely associated with the occurrence of chronic lymphocytic leukemia, being the first research to directly link the deletion or abnormal expression of miRNAs with diseases. More intriguingly, as the second miRNA to be discovered, let‐7 has not only been found to be highly conserved among various species but was also first reported as a tumor suppressor in NSCLC (non‐small cell lung cancer) in 2008.^[^ ^54^ ^]^ This study has offered an important theoretical foundation and research direction for future miRNA‐based cancer therapy. Recently, Xu et al. put forward a novel therapeutic strategy by developing miRNA‐based PROTACs (PROteolysis TArgeting Chimeras) to target the Lin28 protein, restore the expression of the let‐7 family of miRNAs, and thereby inhibit the expression of multiple oncogenes for the treatment of breast cancer.^[^ ^18b^ ^]^ This research not only offers a new therapeutic strategy for cancer but also pioneers a new research direction for the combined application of miRNAs and PROTAC technology. MiRNA‐based PROTACs are expected to become an important tool for the treatment of breast cancer and other cancers in the future, promoting the development of precision medicine and targeting therapy. As of now, the miRNA let‐7 family has achieved remarkable progress in cancer research, encompassing its tumor‐suppressive roles in different cancer types, molecular mechanisms, potential as diagnostic and prognostic markers, as well as therapeutic applications. The research of miRNA in diseases is not merely confined to cancer; there have been numerous studies in various diseases, for instance, the relationship between miR‐21 and myocardial fibrosis.^[^ ^55^ ^]^ In recent years, the research of miRNA in diseases has gradually been transformed into clinical applications, such as early non‐invasive detection of cancer,^[^ ^56^ ^]^ and predicting the survival period of patients through miRNA in exosomes.^[^ ^57^ ^]^

## 5 Application of miRNA for Diagnostic and Therapeutic Strategies

Analysis of global scientific output, research trends, and frontiers in miRNA studies over the last decade revealed that biochemistry and molecular biology have produced the most miRNA‐related papers. The main diseases associated with these studies include cancer, inflammatory bowel disease, neurological disorders, etc.^[^ ^18^, ^58^ ^]^ An analysis of highly cited literature uncovers that much of the research focuses on genetic engineering and targeted therapies, and researchers are active in exploring the clinical applications of miRNA. Many miRNA databases (such as miRBase and TargetScan) and bioinformatics tools (such as miRanda and PicTar) have been developed as a result of high‐throughput sequencing technology to assist researchers in further predicting miRNAs, identifying target genes, and annotating their activities.^[^ ^59^ ^]^ For the time being, miRNA research has progressed from fundamental biological function studies to diagnostic and therapeutic application exploration.

### 5.1 MiRNA‐Based Diagnostic Strategies

Up to now, more than 2000 human miRNAs have been found and play a role in the post‐transcriptional regulation of nearly all cellular functions. As a result, miRNAs have garnered significant attention as potential new tools for the diagnosis of diseases.^[^ ^60^ ^]^ miRNAs are highly conserved regulatory factors with tissue specificity and disease‐related gene expression regulation. Furthermore, miRNAs can be found in a variety of body fluids, including blood, saliva, and urine.^[^ ^58^, ^61^ ^]^ Due to their small hairpin structure, a variety of modifications, and encapsulation within vesicles, miRNAs can resist recognition and degradation by nucleases, as well as withstand extreme conditions such as high temperatures (80 °C), low pH, and freeze‐thaw cycles.^[^ ^62^ ^]^ Because of these features, miRNAs are stable and quantifiable in body fluids, making them ideal non‐invasive biomarkers for early diagnosis and disease progression monitoring.^[^ ^63^ ^]^ Compared to miRNA applications in therapy, the development of diagnostic kits has advanced more smoothly. There are hundreds of miRNA‐based diagnostic clinical trials registered in clinicaltrials.gov.

In 2008, the research by Mitchell, P. S. first systematically identified the presence of miRNA molecules in blood circulation using the qRT‐PCR method.^[^ ^64^ ^]^ Through a series of experiments, it was demonstrated that these circulating miRNAs exhibit high stability in the blood and are capable of resisting degradation by RNA enzymes, making them suitable as non‐invasive biomarkers. In the same year, Rosetta Genomics developed the miRview series, which was the first to help identify the primary site of metastatic tumors by detecting miRNAs using qPCR based custom microarrays.^[^ ^12i^ ^]^ After continuous iterations, the series eventually received FDA (The US Food and Drug Administration) approval in 2013.^[^ ^65^ ^]^ In 2013, Maurice Chan et al.,^[^ ^66^ ^]^ through high‐throughput technology and multi‐stage verification, systematically identified and validated a specific set of miRNA combinations for the identification and prediction of survival in breast cancer patients of different races and regions for the first time. This study provided a reference for the subsequent application of miRNA in the diagnosis of other diseases and promoted the extensive research and application of miRNA as a biomarker.

Recently, in the development of miRNA‐based detection technologies, Hsu et al. have achieved the simultaneous detection of proteins and miRNAs in tumor‐derived exosomes through the development and validation of the Exo‐PROS biosensor.^[^ ^22a^ ^]^ The Exo‐PROS sensor integrates nanomaterials and microfluidic techniques, enabling the concurrent capture and detection of protein and miRNA molecules in exosomes on a single platform. Meanwhile, the sensor employs advanced signal amplification techniques, significantly enhancing detection sensitivity and ensuring the accurate identification of target molecules through specific probes. It realizes a rapid response from sample processing to result acquisition, being suitable for real‐time clinical diagnostic requirements. It also lays the foundation for its commercial application and holds the potential for promotion in clinical practice, providing an innovative solution for the early diagnosis of cancer and precision medicine. In 2024, Wang et al. developed a novel miRNA detection platform based on nanotechnology, integrating a dual‐responsive 3D DNA nanomechanical device and a cascaded hybridization chain reaction (HCR),^[^ ^67^ ^]^ thereby achieving self‐powered, flexible, and highly efficient miRNA detection.

Collectively, the newly developed technology not only outperforms traditional detection methods in terms of sensitivity and specificity but also exhibits portability and multifunctionality, possessing significant application potential in the early diagnosis and real‐time monitoring of cancer.

### 5.2 MiRNA‐Based Therapeutic Strategies

MiRNA has become an important target for clinical treatment research due to its critical regulatory role in disease development and progression. Some of the potential and advantages of miRNA‐based therapeutic strategies may include^[^ ^60^, ^68^ ^]^: 1) Broad impact: miRNAs can regulate the expression of multiple target genes simultaneously, which is beneficial for diseases involving complex networks of dysregulated genes, such as cancer, cardiovascular diseases, and neurological disorders. 2) Targeting undruggable genes: miRNAs can regulate gene expression at the mRNA level, enabling therapeutic intervention even for genes that may be difficult to target directly with traditional small molecules, antibodies, or protein‐based therapies. 3) Predictive biomarkers: miRNAs can serve as biomarkers for disease diagnosis, prognosis, and therapeutic response, helping clinicians track disease progression and response to treatment.^[^ ^69^ ^]^ 4) Versatility in delivery: miRNA‐based therapies can be delivered in various forms, including synthetic mimics or inhibitors. These therapies can be administered through systemic circulation, local injections, or targeted delivery systems (e.g., liposomes, and nanoparticles).^[^ ^70^ ^]^ 5) Reducing the need for gene editing: Unlike gene‐editing strategies (e.g., CRISPR), miRNA‐based therapies don't require permanent changes to the genetic code, which reduces concerns about off‐target effects, unintended genetic modifications, and ethical considerations.^[^ ^71^ ^]^

However, the rapid development of miRNA research has led to a lack of uniform and clear definitions for many basic terms, and the complexity of miRNA regulatory networks has hindered the swift clinical application of miRNA‐related therapies.^[^ ^18^, ^60^, ^72^ ^]^ We have updated the currently active, completed, terminated miRNA‐based therapeutic clinical trials based on clinicaltrials.gov and literature reports^[^ ^35^, ^60^, ^72^ ^]^ (**Table** **1**). So far, there are only 20 more miRNA‐based therapeutic clinical trials and most of them have failed. There are only 3 in the active stage and no phase III study. Here are some examples of failed studies.

**Table 1:** The clinical trials of miRNA inhibitor or miRNA mimic.

NCT Number	Grug name	Target miRNA	Conditions	Phases(Enrollment)	Study Status
NCT02612662	AZD4076 (RG‐125)	miR‐103/107	Non‐alcoholic Steatohepatitis	I (40)	Active, not recruiting
NCT04120493	rAAV5‐miHTT (AMT‐130)	Pri‐miR‐451 backbone	Huntington disease	I/II (37)	Active, recruiting
NCT00688012	Miravirsen (SPC3649)	miR‐122	Hepatitis C	I (64)	Completed (2009)
NCT01200420	Miravirsen (SPC3649)	miR‐122	Hepatitis C	II (38)	Completed (2012)
NCT02369198	TargomiRs (MesomiR1)	miR‐16 mimic	Malignant pleural mesothelioma; NSCLC	I (27)	Completed (2017)
NCT03603431	MRG‐110 (S95010)	miR‐92a	Healthy volunteer	I (42)	Completed (2019)
NCT03373786	Lademirsen (RG‐012)	miR‐21	Alport syndrome	I (4)	Completed (2019)
NCT02826525	AZD4076 (RG‐125)	miR‐103/107	Type 2 diabetic subjects with non‐alcoholic fatty liver disease	I (14)	Completed (2019)
NCT03601052	Remlarsen (MRG201)	miR‐29 mimic	Keloid	II (14)	Completed (2020)
NCT04045405	CDR132L	miR‐132	Heart failure	Ib (28)	Completed (2020)
NCT05350969	CDR132L	miR‐132	Heart failure	II (294)	Active, not recruiting
NCT03494712	MRG‐110	miR‐92a	Cardiovascular diseases	I (49)	Completed (2020)
NCT04536688	RGLS4326	miR‐17	Polycystic kidney disease; Autosomal dominant	I (19)	Completed (2021)
NCT04811898	LNA‐i‐miR‐221	miR‐221	Multiple myeloma, Refractory; Hepatocarcinoma; Advanced solid tumor	I (17)	Completed (2021)
NCT01829971	MRX34 (miR‐34a Mimic)	miR‐34a	Liver cancer; SCLC; Lymphoma; Melanoma; RCC; NSCLC	I (155)	Terminated (2017)
NCT03713320	Cobomarsen (MRG106)	miR‐155	Lymphoma; Mycosis fungoides;	II (37)	Terminated (2020)
NCT02580552	Cobomarsen (MRG106)	miR‐155	Lymphoma; Mycosis fungoides	I (66)	Terminated (2020)
NCT03837457	Cobomarsen (MRG106)	miR‐155	Lymphoma, Mycosis Fungoides	II (8)	Terminated (2020)
NCT02855268	lademirsen (SAR339375)	miR‐21	Alport syndrome	II (43)	Terminated (2023)

Note: All the information is taken from https://clinicaltrials.gov. RCC: Renal Cell Carcinoma

**Miravirsen** (SPC3649), the first miRNA‐targeted drug to enter clinical trials,^[^ ^73^ ^]^ is an anti‐miRNA oligonucleotide targeting miR‐122^[^ ^74^ ^]^ for hepatitis C treatment developed by Santaris Pharma A/S. It entered phase 1 clinical trials in 2008 and phase 2 in 2010, respectively. It demonstrated good tolerance and no serious adverse events, revealing its effectiveness in inhibiting miR‐122. Eventually, it was not further developed after the Phase 2 clinical trials due to the occurrence of hyperbilirubinemia and a shift in market demand resulting in low efficacy.^[^ ^75^ ^]^

**MRX34**, as the first miRNA mimic‐based therapeutic drug for multiple cancer treatments and entering clinical trials, is based on the mimic of miR‐34a carried by lipid nanoparticles to treat various advanced solid tumors starting in 2013 and ending in 2017.^[^ ^76^ ^]^ Although it showed some efficacy with 3 patients having RP (partial response) and 16 having SD (stable disease) lasting for more than 4 cycles during the trial stage, it was forced to be suspended due to serious drug‐related SAEs (serious adverse events). The major failure reasons are severe immune‐mediated toxicities such as fever, back/neck pain, nausea, sepsis, hypoxia, cytokine release syndrome, and multiple organ failure that caused four patients' deaths in the expansion cohorts. The other possible reasons are the delivery system of liposome nanoparticles^[^ ^77^ ^]^ and the multi‐target genes of miRNA‐34^[^ ^72^, ^78^ ^]^ led to the possibility that MRX34 affected multiple biological pathways in the body, increasing the risk of side effects and endangering the lives of patients.

**TargomiRs (MesomiR1),** the second miRNA mimic‐based (miR‐16 minic) therapy, entered Phase 1 clinical trials in 2014 ‐2017 with a novel delivery system for patients with recurrent malignant pleural mesothelioma and NSCLC.^[^ ^79^ ^]^ Its delivery system consists of minicells with miR‐16 based mimics produced by genetically engineered Escherichia coli. As minicells lack a nucleus, they possess low immunogenicity and toxicity, reducing side effects on normal cells and tissues. The Phase 1 result shows that one (5%) had a partial response, 15 (68%) had stable disease, and 6 (27%) had progressive disease. The major toxicities are infusion‐related inflammatory symptoms and coronary ischemia. Although the microcell vector employed by TargomiRs has excellent targeting and biocompatibility, how to further enhance the delivery efficiency and ensure the efficient absorption and functional restoration of miR‐16 mimics in tumor cells remains a research priority. Combining TargomiRs with chemotherapy or immune checkpoint inhibitors may be one potential way for a randomized phase 2 clinical trial study in the future.

The active clinical trials are AZD4076 for inhibiting miR‐103/107, CDR132L for anti‐miR‐132,^[^ ^80^ ^]^ and AMT130 for targeting pri‐miR‐451 backbone (Table 1). There are no persistent serious adverse events for AMT130 in Huntington's disease.^[^ ^81^ ^]^

The development process of these drugs fully demonstrates the significant challenges faced in the development of miRNA‐based drugs, such as deterring the miRNAs accurately targeting its mRNAs without off‐target effects, reducing drug toxicity, especially immunogenic‐related reactions, enhancing delivery system, and correct dosing.^[^ ^60^, ^72^, ^82^ ^]^ With these critical obstacles, therefore, at present, no miRNA‐based drug has been approved by FDA.

As with a new therapeutic modality, the development and application of miRNA/RNAi‐based therapy/clinical trials raise ethical and regulatory issues.^[^ ^82^, ^83^ ^]^ The four principles of biomedical ethics,^[^ ^83c^ ^]^ e, g. autonomous, beneficence, nonmaleficence, and justice, are the basic regulations. A clear guideline for the preclinical and clinical evaluation of miRNA therapeutics should be established. The off‐target effects and immunogenic‐related toxicity should be addressed as potential risks. Despite hindrances, the encouraging progress in optimized designs for miRNA‐based therapeutics promises their increasingly impactful role in future medicine.

## 6 Challenges and Future Directions of miRNA Research and Application

### 6.1 Challenges of miRNA Research and Application

The future of miRNA research holds immense potential for advancing our understanding of gene regulation and developing novel diagnosis and therapeutic approaches. However, several significant challenges or dilemmas remain.^[^ ^1^, ^60^, ^72^ ^]^

1. The accurate identification of miRNA targets. The partial complementarity between miRNAs and their targets makes computational prediction difficult, necessitating rigorous experimental validation.^[^ ^84^ ^]^ High‐throughput methods such as CLIP‐Seq (cross‐linking immunoprecipitation coupled with sequencing) have been developed to identify miRNA binding sites, but these techniques require further refinement to improve their resolution and reliability.^[^ ^85^ ^]^
2. The functional redundancy and compensation miRNA. MiRNAs often exhibit functional redundancy, where multiple miRNAs can regulate the same target, or a single miRNA can regulate multiple targets. This redundancy complicates the interpretation of loss‐ or gain‐of‐function studies.
3. The miRNA‐based therapeutic related issues including efficient delivery systems, off‐target effects, and immunogenic‐related toxicity. Continued interdisciplinary research combining biology, bioinformatics, and clinical sciences will be essential to overcome these hurdles and unlock the full potential of miRNAs.

### 6.2 Future Directions of miRNA Research and Application

The field of miRNA research is rapidly evolving, while several future directions can be anticipated:

1. Understanding the complex molecules regulatory networks and mechanisms involving miRNAs.^[^ ^86^ ^]^ Integrating miRNA data with other omics data (e.g., genomics, proteomics) and deep learning models will elucidate the multilayered regulatory roles of miRNAs in different cellular processes and diseases.^[^ ^18^, ^86^, ^87^ ^]^
2. MiRNA editing and modifications: Advances in genome editing technologies, such as CRISPR/Cas9, offer the potential to edit miRNA genes directly.^[^ ^88^ ^]^ Understanding the mechanisms of miRNA editing and post‐transcriptional modifications could be used to correct miRNA dysregulation in various diseases. The adenosine‐to‐inosine (A‐to‐I) editing can significantly alter miRNA function, target specificity, and stability.^[^ ^89^ ^]^ Delving deeper into the enzymes and pathways responsible for these modifications may open new therapeutic avenues and provide insights into disease mechanisms. For instance, aberrant editing events have been linked to cancer and neurological disorders, highlighting their potential as therapeutic targets.^[^ ^51^, ^90^ ^]^
3. Single‐cell miRNA study: The development of techniques to analyze miRNA expression at the single‐cell level will be pivotal.^[^ ^91^ ^]^ Single‐cell RNA sequencing (scRNA‐seq) technologies have already provided breakthroughs in understanding cellular heterogeneity in tissues. Adapting these techniques for miRNA profiling will help uncover cell‐type‐specific miRNA functions and their role in developmental processes and disease etiology. This approach will also enable the identification of rare cell populations that play critical roles in disease progression, offering new targets for therapeutic intervention.^[^ ^91b^ ^]^
4. MiRNA biomarkers: The identification of miRNA signatures as biomarkers for various diseases is a rapidly growing area.^[^ ^61^, ^86^ ^]^ Future research should aim at validating these biomarkers in large, diverse populations to improve their clinical applicability. This involves developing standardized protocols for sample collection, miRNA extraction, and quantification.^[^ ^92^ ^]^ Additionally, integrating multi‐omics data, including transcriptomics, proteomics, and metabolomics, can enhance the specificity and sensitivity of miRNA‐based diagnostics, aiding in early disease detection and personalized medicine.^[^ ^93^ ^]^
5. MiRNA‐based therapies: Research into miRNA‐based therapeutics is promising but requires comprehensive studies to ensure efficacy and safety.^[^ ^76^, ^94^ ^]^ Future research should focus on developing miRNA drugs that are more specific, stable, and possess low toxicity, along with tools capable of targeted delivery. CRISPR/Cas technology to target miRNAs in disease therapy has been explored and needs further study.^[^ ^71^ ^]^ Clinical trials and rigorous validation studies will be required to transition these therapeutics from bench to bedside.^[^ ^86^, ^90^ ^]^

## 7 Conclusion

Since the discovery of miRNAs, scientists have spent decades unraveling their biogenesis, functions, and mechanisms of action, while also attempting to apply them in the diagnosis and treatment of diseases. The future of miRNA research holds immense potential for advancing our understanding of gene regulation networks and developing novel diagnostics and therapeutic approaches. Continued interdisciplinary research combining biology, bioinformatics, and clinical sciences will be essential to overcome these hurdles and unlock the full potential of miRNAs.^[^ ^95^ ^]^

## Conflict of Interest

The authors declare no conflict of interest.

## References

[1] a) E. C. Lai , Nat. Genet. 2002, 30, 363; 11896390 10.1038/ng865

[2] B. J. Reinhart , F. J. Slack , M. Basson , A. E. Pasquinelli , J. C. Bettinger , A. E. Rougvie , H. R. Horvitz , G. Ruvkun , Nature 2000, 403, 901. 10706289 10.1038/35002607

[3] a) W. Peng , Z. He , W. Dai , W. Lan , Briefings in bioinformatics 2023, 25, bbad524; 38243694 10.1093/bib/bbad524 PMC10796254

[4] The Nobel Prize in Physiology or Medicine, https://www.nobelprize.org/prizes/medicine/2024/summary/, (accessed: October 2024).

[5] M. Lagos‐Quintana , R. Rauhut , W. Lendeckel , T. Tuschl , Science (New York, N.Y.) 2001, 294, 853. 11679670 10.1126/science.1064921

[6] R. C. Lee , R. L. Feinbaum , V. Ambros , Cell 1993, 75, 843. 8252621 10.1016/0092-8674(93)90529-y

[7] B. Wightman , I. Ha , G. Ruvkun , Cell 1993, 75, 855. 8252622 10.1016/0092-8674(93)90530-4

[8] a) N. C. Lau , L. P. Lim , E. G. Weinstein , D. P. Bartel , Science (New York, N.Y.) 2001, 294, 858; 11679671 10.1126/science.1065062

[9] A. Grishok , A. E. Pasquinelli , D. Conte , N. Li , S. Parrish , I. Ha , D. L. Baillie , A. Fire , G. Ruvkun , C. C. Mello , Cell 2001, 106, 23. 11461699 10.1016/s0092-8674(01)00431-7

[10] S. Griffiths‐Jones , Nucleic acids research (Database issue) 2004, 32, D109. 10.1093/nar/gkh023 PMC308757 14681370

[11] G. Meister , M. Landthaler , A. Patkaniowska , Y. Dorsett , G. Teng , T. Tuschl , Mol. Cell 2004, 15, 185. 15260970 10.1016/j.molcel.2004.07.007

[12] a) R. Yi , Y. Qin , I. G. Macara , B. R. Cullen , Genes Dev. 2003, 17, 3011; 14681208 10.1101/gad.1158803 PMC305252

[13] Lasker Foundation: For discoveries that revealed an unanticipated world of tiny RNAs that regulate gene function in plants and animals, https://laskerfoundation.org/winners/tiny‐rnas‐that‐regulate‐gene‐function/ (accessed: September 2008).

[14] T. Treiber , N. Treiber , U. Plessmann , S. Harlander , J. L. Daiß , N. Eichner , G. Lehmann , K. Schall , H. Urlaub , G. Meister , Mol. Cell 2017, 66, 270. 28431233 10.1016/j.molcel.2017.03.014

[15] a) M. Xiao , J. Li , W. Li , Y. Wang , F. Z. Wu , Y. P. Xi , L. Zhang , C. Ding , H. B. Luo , Y. Li , L. Peng , L. P. Zhao , S. L. Peng , Y. Xiao , S. H. Dong , J. Cao , W. Q. Yu , Rna Biol 2017, 14, 1326; 26853707 10.1080/15476286.2015.1112487 PMC5711461

[16] D. Jee , J. S. Yang , S. M. Park , D. T. Farmer , J. Wen , T. Chou , A. Chow , M. T. McManus , M. G. Kharas , E. C. Lai , Mol. Cell 2018, 69, 265. 29351846 10.1016/j.molcel.2017.12.027 PMC5824974

[17] X. Y. Qiu , L. Y. Zhu , C. S. Zhu , J. X. Ma , T. Hou , X. M. Wu , S. S. Xie , L. Min , D. A. Tan , D. Y. Zhang , L. Zhu , ACS Synth. Biol. 2018, 7, 807. 29486117 10.1021/acssynbio.7b00446

[18] a) N. Perdikopanis , G. K. Georgakilas , D. Grigoriadis , V. Pierros , I. Kavakiotis , P. Alexiou , A. Hatzigeorgiou , Nucleic Acids Res. 2021, 49, D151; 33245765 10.1093/nar/gkaa1060 PMC7778932

[19] Y. Y. Lee , H. Lee , H. Kim , V. N. Kim , S. H. Roh , Nature 2023, 615, 331. 36813958 10.1038/s41586-023-05723-3

[20] T. Hennig , A. B. Prusty , B. B. Kaufer , A. W. Whisnant , M. Lodha , A. Enders , J. Thomas , F. Kasimir , A. Grothey , T. Klein , S. Herb , C. Jürges , M. Sauer , U. Fischer , T. Rudel , G. Meister , F. Erhard , L. Dölken , B. K. Prusty , Nature 2022, 605, 539. 35508655 10.1038/s41586-022-04667-4

[21] A. Fernández‐Pato , A. Virseda‐Berdices , S. Resino , P. Ryan , O. Martínez‐González , F. Pérez‐García , M. Martin‐Vicente , D. Valle‐Millares , O. Brochado‐Kith , R. Blancas , A. Martínez , F. C. Ceballos , S. Bartolome‐Sánchez , E. J. Vidal‐Alcántara , D. Alonso , N. Blanca‐López , I. R. Martinez‐Acitores , L. Martin‐Pedraza , M. Jiménez‐Sousa , A. Fernández‐Rodríguez , Emerging microbes & infections 2022, 11, 676. 35130828 10.1080/22221751.2022.2038021 PMC8890551

[22] a) C. C. Hsu , Y. Yang , E. Kannisto , X. Zeng , G. Yu , S. K. Patnaik , G. K. Dy , M. E. Reid , Q. Gan , Y. Wu , ACS Nano 2023, 17, 8108; 37129374 10.1021/acsnano.2c10970 PMC10266547

[23] a) A. C. Partin , K. Zhang , B. C. Jeong , E. Herrell , S. Li , W. Chiu , Y. Nam , Mol. Cell 2020, 78, 411; 32220646 10.1016/j.molcel.2020.02.016 PMC7214211

[24] a) G. Hutvágner , J. McLachlan , A. E. Pasquinelli , E. Bálint , T. Tuschl , P. D. Zamore , Science (New York, N.Y.) 2001, 293, 834; 11452083 10.1126/science.1062961

[25] E. Yigit , P. J. Batista , Y. Bei , K. M. Pang , C. C. Chen , N. H. Tolia , L. Joshua‐Tor , S. Mitani , M. J. Simard , C. C. Mello , Cell 2006, 127, 747. 17110334 10.1016/j.cell.2006.09.033

[26] C. Ender , A. Krek , M. R. Friedländer , M. Beitzinger , L. Weinmann , W. Chen , S. Pfeffer , N. Rajewsky , G. Meister , Mol. Cell 2008, 32, 519. 19026782 10.1016/j.molcel.2008.10.017

[27] J. E. Babiarz , J. G. Ruby , Y. Wang , D. P. Bartel , R. Blelloch , Genes Dev. 2008, 22, 2773. 18923076 10.1101/gad.1705308 PMC2569885

[28] H. P. Bogerd , H. W. Karnowski , X. Cai , J. Shin , M. Pohlers , B. R. Cullen , Mol. Cell 2010, 37, 135. 20129062 10.1016/j.molcel.2009.12.016 PMC2818755

[29] S. Cheloufi , C. O. Dos Santos , M. M. Chong , G. J. Hannon , Nature 2010, 465, 584. 20424607 10.1038/nature09092 PMC2995450

[30] E. P. Murchison , J. F. Partridge , O. H. Tam , S. Cheloufi , G. J. Hannon , Proc. Natl. Acad. Sci. USA 2005, 102, 12135. 16099834 10.1073/pnas.0505479102 PMC1185572

[31] a) S. Bagga , J. Bracht , S. Hunter , K. Massirer , J. Holtz , R. Eachus , A. E. Pasquinelli , Cell 2005, 122, 553; 16122423 10.1016/j.cell.2005.07.031

[32] J. R. Lytle , T. A. Yario , J. A. Steitz , Proc. Natl. Acad. Sci. USA 2007, 104, 9667. 17535905 10.1073/pnas.0703820104 PMC1887587

[33] A. M. Eiring , J. G. Harb , P. Neviani , C. Garton , J. J. Oaks , R. Spizzo , S. Liu , S. Schwind , R. Santhanam , C. J. Hickey , H. Becker , J. C. Chandler , R. Andino , J. Cortes , P. Hokland , C. S. Huettner , R. Bhatia , D. C. Roy , S. A. Liebhaber , M. A. Caligiuri , G. Marcucci , R. Garzon , C. M. Croce , G. A. Calin , D. Perrotti , Cell 2010, 140, 652. 20211135 10.1016/j.cell.2010.01.007 PMC2924756

[34] a) Q. Jing , S. Huang , S. Guth , T. Zarubin , A. Motoyama , J. Chen , F. Di Padova , S. C. Lin , H. Gram , J. Han , Cell 2005, 120, 623; 15766526 10.1016/j.cell.2004.12.038

[35] a) A. Kilikevicius , G. Meister , D. R. Corey , Nucleic Acids Res. 2022, 50, 617; 34967419 10.1093/nar/gkab1256 PMC8789053

[36] a) M. V. Iorio , C. M. Croce , Carcinogenesis 2012, 33, 1126; 22491715 10.1093/carcin/bgs140 PMC3514864

[37] S. Jiang , L. F. Zhang , H. W. Zhang , S. Hu , M. H. Lu , S. Liang , B. Li , Y. Li , D. Li , E. D. Wang , M. F. Liu , EMBO J. 2012, 31, 1985. 22354042 10.1038/emboj.2012.45 PMC3343331

[38] T. Ito , S. Yagi , M. Yamakuchi , Biochem. Biophys. Res. Commun. 2010, 398, 735. 20627091 10.1016/j.bbrc.2010.07.012

[39] S. Ye , L. Wei , Y. Jiang , Y. Yuan , Y. Zeng , L. Zhu , F. Xiao , J. Hazard. Mater. 2024, 465, 133362. 38157813 10.1016/j.jhazmat.2023.133362

[40] M. Yuan , Z. Cao , Q. Li , R. Liu , J. Wang , W. Xue , Q. Lyu , Metabolism: clinical and experimental 2024, 158, 155959. 38942170 10.1016/j.metabol.2024.155959

[41] a) R. Wang , C. Zhang , W. Guan , Q. Yang , Neoplasma 2023, 70, 566; 37789781 10.4149/neo_2023_230410N196

[42] P. Xu , S. Y. Vernooy , M. Guo , B. A. Hay , Current biology : CB 2003, 13, 790. 12725740 10.1016/s0960-9822(03)00250-1

[43] A. Cimmino , G. A. Calin , M. Fabbri , M. V. Iorio , M. Ferracin , M. Shimizu , S. E. Wojcik , R. I. Aqeilan , S. Zupo , M. Dono , L. Rassenti , H. Alder , S. Volinia , C. G. Liu , T. J. Kipps , M. Negrini , C. M. Croce , Proc. Natl. Acad. Sci. USA 2005, 102, 13944. 16166262 10.1073/pnas.0506654102 PMC1236577

[44] C. Z. Chen , L. Li , H. F. Lodish , D. P. Bartel , Science (New York, N.Y.) 2004, 303, 83. 14657504 10.1126/science.1091903

[45] C. Esau , X. Kang , E. Peralta , E. Hanson , E. G. Marcusson , L. V. Ravichandran , Y. Sun , S. Koo , R. J. Perera , R. Jain , N. M. Dean , S. M. Freier , C. F. Bennett , B. Lollo , R. Griffey , The Journal of biological chemistry 2004, 279, 52361. 15504739 10.1074/jbc.C400438200

[46] a) F. Sepulveda , C. Mayorga‐Lobos , K. Guzman , E. Duran‐Jara , L. Lobos‐Gonzalez , Int. J. Mol. Sci. 2023, 24, 13085; 37685891 10.3390/ijms241713085 PMC10487525

[47] N. Kosaka , H. Iguchi , Y. Yoshioka , F. Takeshita , Y. Matsuki , T. Ochiya , The Journal of biological chemistry 2010, 285, 17442. 20353945 10.1074/jbc.M110.107821 PMC2878508

[48] a) M. Ueta , H. Nishigaki , S. Komai , K. Mizushima , R. Tamagawa‐Mineoka , Y. Naito , N. Katoh , C. Sotozono , S. Kinoshita , Front. Genet. 2022, 13, 1025539; 36685889 10.3389/fgene.2022.1025539 PMC9858567

[49] K. D. Taganov , M. P. Boldin , K. J. Chang , D. Baltimore , Proc. Natl. Acad. Sci. USA 2006, 103, 12481. 16885212 10.1073/pnas.0605298103 PMC1567904

[50] S. Ressel , S. Kumar , J. R. Bermúdez‐Barrientos , K. Gordon , J. Lane , J. Wu , C. Abreu‐Goodger , J. Schwarze , A. H. Buck , Nucleic Acids Res. 2024, 52, 4872. 38412296 10.1093/nar/gkae116 PMC11109944

[51] a) P. Wang , G. Xiong , D. Zeng , J. Zhang , L. Ge , L. Liu , X. Wang , Y. Hu , BMC Genomics 2022, 23, 801; 36471254 10.1186/s12864-022-09029-y PMC9721069

[52] A. J. Giraldez , R. M. Cinalli , M. E. Glasner , A. J. Enright , J. M. Thomson , S. Baskerville , S. M. Hammond , D. P. Bartel , A. F. Schier , Science (New York, N.Y.) 2005, 308, 833. 15774722 10.1126/science.1109020

[53] a) L. Zu , J. He , N. Zhou , Q. Tang , M. Liang , S. Xu , Cell Death Dis. 2023, 14, 798; 38057344 10.1038/s41419-023-06286-x PMC10700602

[54] M. S. Kumar , S. J. Erkeland , R. E. Pester , C. Y. Chen , M. S. Ebert , P. A. Sharp , T. Jacks , Proc. Natl. Acad. Sci. USA 2008, 105, 3903. 18308936 10.1073/pnas.0712321105 PMC2268826

[55] a) T. Thum , C. Gross , J. Fiedler , T. Fischer , S. Kissler , M. Bussen , P. Galuppo , S. Just , W. Rottbauer , S. Frantz , M. Castoldi , J. Soutschek , V. Koteliansky , A. Rosenwald , M. A. Basson , J. D. Licht , J. T. Pena , S. H. Rouhanifard , M. U. Muckenthaler , T. Tuschl , G. R. Martin , J. Bauersachs , S. Engelhardt , Nature 2008, 456, 980; 19043405 10.1038/nature07511

[56] W. Shi , T. Wartmann , S. Accuffi , S. Al‐Madhi , A. Perrakis , C. Kahlert , A. Link , M. Venerito , V. Keitel‐Anselmino , C. Bruns , R. S. Croner , Y. Zhao , U. D. Kahlert , Br. J. Cancer 2024, 130, 125. 37950093 10.1038/s41416-023-02488-4 PMC10781694

[57] a) Y. Pan , X. Xu , T. Luo , S. Yang , D. Zhou , Y. Zeng , Biomed Res. Int. 2022, 2022, 5170261; 36312858 10.1155/2022/5170261 PMC9615554

[58] a) Z. Alkhazaali‐Ali , S. Sahab‐Negah , A. R. Boroumand , J. Tavakol‐Afshari , Biomed. Pharmacother. 2024, 177, 116899; 38889636 10.1016/j.biopha.2024.116899

[59] a) L. Chen , L. Heikkinen , C. Wang , Y. Yang , H. Sun , G. Wong , Brief Bioinform 2019, 20, 1836; 29982332 10.1093/bib/bby054 PMC7414524

[60] a) C. Diener , A. Keller , E. Meese , Trends Genet. 2022, 38, 613; 35303998 10.1016/j.tig.2022.02.006

[61] a) M. Prasad , R. Sekar , M. D. L. Priya , S. R. Varma , M. I. Karobari , Diagn Pathol 2024, 19, 147; 39548527 10.1186/s13000-024-01575-1 PMC11568613

[62] a) Z. Li , D. Chen , Q. Wang , H. Tian , M. Tan , D. Peng , Y. Tan , J. Zhu , W. Liang , L. Zhang , Forensic science international. Genetics 2021, 55, 102567; 34403952 10.1016/j.fsigen.2021.102567

[63] B. Ma , S. Wang , W. Wu , P. Shan , Y. Chen , J. Meng , L. Xing , J. Yun , L. Hao , X. Wang , S. Li , Y. Guo , Biomedicine & pharmacotherapy = Biomedecine & pharmacotherapie 2023, 162, 114672. 37060662 10.1016/j.biopha.2023.114672

[64] P. S. Mitchell , R. K. Parkin , E. M. Kroh , B. R. Fritz , S. K. Wyman , E. L. Pogosova‐Agadjanyan , A. Peterson , J. Noteboom , K. C. O'Briant , A. Allen , D. W. Lin , N. Urban , C. W. Drescher , B. S. Knudsen , D. L. Stirewalt , R. Gentleman , R. L. Vessella , P. S. Nelson , D. B. Martin , M. Tewari , Proc. Natl. Acad. Sci. USA 2008, 105, 10513. 18663219 10.1073/pnas.0804549105 PMC2492472

[65] R. G. Ltd , Announces the Availability of miRview(R) mets(2), a Second Generation Version of miRview(R) mets 2010, https://www.biospace.com/rosetta‐genomics‐ltd‐announces‐the‐availability‐of‐mirview‐r‐mets‐2‐a‐second‐generation‐version‐of‐mirview‐r‐mets (accessed: September 2008).

[66] M. Chan , C. S. Liaw , S. M. Ji , H. H. Tan , C. Y. Wong , A. A. Thike , P. H. Tan , G. H. Ho , A. S. Lee , American Association for Cancer Research 2013, 19, 4477. 10.1158/1078-0432.CCR-12-3401 23797906

[67] F. Wang , C. Zhang , S. Deng , Y. Jiang , P. Zhang , H. Yang , L. Xiang , Y. Lyu , R. Cai , W. Tan , Biosens. Bioelectron. 2024, 252, 116149. 38394701 10.1016/j.bios.2024.116149

[68] a) M. Y. Shah , A. Ferrajoli , A. K. Sood , G. Lopez‐Berestein , G. A. Calin , EBioMedicine 2016, 12, 34; 27720213 10.1016/j.ebiom.2016.09.017 PMC5078622

[69] W. Jelski , J. Mroczko , S. Okrasinska , B. Mroczko , Cancers (Basel) 2024, 16, 3809. 39594763 10.3390/cancers16223809 PMC11593317

[70] M. Pagoni , C. Cava , D. C. Sideris , M. Avgeris , V. Zoumpourlis , I. Michalopoulos , N. Drakoulis , J. Pers. Med. 2023, 13, 1586. 38003902 10.3390/jpm13111586 PMC10672431

[71] B. M. Hussen , M. F. Rasul , S. R. Abdullah , H. J. Hidayat , G. S. H. Faraj , F. A. Ali , A. Salihi , A. Baniahmad , S. Ghafouri‐Fard , M. Rahman , M. C. Glassy , W. Branicki , M. Taheri , Mil Med Res 2023, 10, 32. 37460924 10.1186/s40779-023-00468-6 PMC10351202

[72] a) S. Zhang , Z. J. Cheng , Y. N. Wang , T. Y. Han , Drug Des Dev Ther 2021, 15, 721; 10.2147/DDDT.S288859 PMC7910153 33654378

[73] a) R. E. Lanford , E. S. Hildebrandt‐Eriksen , A. Petri , R. Persson , M. Lindow , M. E. Munk , S. Kauppinen , H. Ørum , Science (New York, N.Y.) 2010, 327, 198; 19965718 10.1126/science.1178178 PMC3436126

[74] L. F. R. Gebert , M. A. E. Rebhan , S. E. M. Crivelli , R. Denzler , M. Stoffel , J. Hall , Nucleic Acids Res. 2014, 42, 609. 24068553 10.1093/nar/gkt852 PMC3874169

[75] M. H. van der Ree , J. M. de Vree , F. Stelma , S. Willemse , M. van der Valk , S. Rietdijk , R. Molenkamp , J. Schinkel , A. van Nuenen , U. Beuers , S. Hadi , M. Harbers , E. van der Veer , K. Liu , J. Grundy , A. K. Patick , A. Pavlicek , J. Blem , M. Huang , P. Grint , S. Neben , N. W. Gibson , N. A. Kootstra , H. W. Reesink , Lancet 2017, 389, 709. 28087069 10.1016/S0140-6736(16)31715-9

[76] D. S. Hong , Y. K. Kang , M. Borad , J. Sachdev , S. Ejadi , H. Y. Lim , A. J. Brenner , K. Park , J. L. Lee , T. Y. Kim , S. Shin , C. R. Becerra , G. Falchook , J. Stoudemire , D. Martin , K. Kelnar , H. Peltier , V. Bonato , A. G. Bader , S. Smith , S. Kim , V. O'Neill , M. S. Beg , Br. J. Cancer 2020, 122, 1630. 32238921 10.1038/s41416-020-0802-1 PMC7251107

[77] M. S. Beg , A. J. Brenner , J. Sachdev , M. Borad , Y. K. Kang , J. Stoudemire , S. Smith , A. G. Bader , S. Kim , D. S. Hong , Invest New Drug 2017, 35, 180. 10.1007/s10637-016-0407-y PMC5893501 27917453

[78] a) M. A. Cortez , C. Ivan , D. Valdecanas , X. H. Wang , H. J. Peltier , Y. P. Ye , L. Araujo , D. P. Carbone , K. Shilo , D. K. Giri , K. Kelnar , D. Martin , R. Komaki , D. R. Gomez , S. Krishnan , G. A. Calin , A. G. Bader , J. W. Welsh , Jnci‐J Natl Cancer I 2016, 108, djv303; 10.1093/jnci/djv303 PMC4862407 26577528

[79] N. van Zandwijk , N. Pavlakis , S. C. Kao , A. Linton , M. J. Boyer , S. Clarke , Y. Huynh , A. Chrzanowska , M. J. Fulham , D. L. Bailey , W. A. Cooper , L. Kritharides , L. Ridley , S. T. Pattison , J. MacDiarmid , H. Brahmbhatt , G. Reid , The Lancet. Oncology 2017, 18, 1386. 28870611 10.1016/S1470-2045(17)30621-6

[80] J. Bauersachs , S. D. Solomon , S. D. Anker , I. Antorrena‐Miranda , S. Batkai , J. Viereck , S. Rump , G. Filippatos , U. Granzer , P. Ponikowski , R. A. de Boer , O. Vardeny , W. Hauke , T. Thum , Eur. J. Heart Fail 2024, 26, 674. 38269451 10.1002/ejhf.3139

[81] C. Estevez‐Fraga , S. J. Tabrizi , E. J. Wild , J. Huntingtons Dis. 2024, 13, 1. 38489195 10.3233/JHD-240017 PMC11091610

[82] a) A. A. Seyhan , Int. J. Mol. Sci. 2024, 25, 1469;38338746

[83] a) P. Garg , G. Singhal , S. Pareek , P. Kulkarni , D. Horne , A. Nath , R. Salgia , S. S. Singhal , Biochim. Biophys. Acta. Rev. Cancer 2024, 1880, 189233; 39638158 10.1016/j.bbcan.2024.189233

[84] K. Uthayopas , A. G. C. de Sa , A. Alavi , D. E. V. Pires , D. B. Ascher , Comput. Struct. Biotechnol. J. 2024, 23, 3030. 39175797 10.1016/j.csbj.2024.06.030 PMC11340604

[85] a) E. Y. Rykova , N. I. Ershov , A. O. Degtyareva , L. O. Bryzgalov , E. L. Lushnikova , Bull. Exp. Biol. Med. 2024, 176, 595; 38724816 10.1007/s10517-024-06074-3

[86] a) C. Cui , B. Zhong , R. Fan , Q. Cui , Nucleic Acids Res. 2024, 52, D1327; 37650649 10.1093/nar/gkad717 PMC10767894

[87] a) F. Kern , L. Krammes , K. Danz , C. Diener , T. Kehl , O. Kuchler , T. Fehlmann , M. Kahraman , S. Rheinheimer , E. Aparicio‐Puerta , S. Wagner , N. Ludwig , C. Backes , H. P. Lenhof , H. von Briesen , M. Hart , A. Keller , E. Meese , Nucleic Acids Res. 2021, 49, 127; 33305319 10.1093/nar/gkaa1161 PMC7797041

[88] M. K. Prajapat , A. G. Maria , J. A. Vidigal , Nucleic Acids Res. 2024, 53, gkae1138. 10.1093/nar/gkae1138 PMC11724307 39673524

[89] Q. Yin , Z. Qu , R. Mathew , L. Zeng , Z. Du , Y. Xue , D. Liu , X. Zheng , Cell Biochem. Funct. 2024, 42, e3996. 38561942 10.1002/cbf.3996

[90] C. R. Alarcon , H. Lee , H. Goodarzi , N. Halberg , S. F. Tavazoie , Nature 2015, 519, 482. 25799998 10.1038/nature14281 PMC4475635

[91] a) Y. Yao , Z. Wang , Y. Chen , L. Liu , L. Wang , G. Yi , Y. Yang , D. Wang , K. Li , Z. Tang , Genes Dis. 2023, 10, 359; 37223503 10.1016/j.gendis.2022.04.012 PMC10201586

[92] a) B. Yaylak , B. Akgul , Methods Mol. Biol. 2022, 2257, 33; 34432272 10.1007/978-1-0716-1170-8_2

[93] K. S. Akshay Pramod Ware , B. Paul , Funct. Integr. Genomics 2024, 24, 133. 39085735 10.1007/s10142-024-01410-2 PMC11291601

[94] a) J. Taubel , W. Hauke , S. Rump , J. Viereck , S. Batkai , J. Poetzsch , L. Rode , H. Weigt , C. Genschel , U. Lorch , C. Theek , A. A. Levin , J. Bauersachs , S. D. Solomon , T. Thum , Eur. Heart J. 2021, 42, 178; 33245749 10.1093/eurheartj/ehaa898 PMC7954267

[95] a) N. D. Akingbesote , B. P. Leitner , D. G. Jovin , R. Desrouleaux , D. Owusu , W. Zhu , Z. Li , M. N. Pollak , R. J. Perry , Elife 2023, 12, e78335; 37219930 10.7554/eLife.78335 PMC10205083

